# Greenbug (*Schizaphis graminum*) herbivory significantly impacts protein and phosphorylation abundance in switchgrass (*Panicum virgatum*)

**DOI:** 10.1038/s41598-020-71828-8

**Published:** 2020-09-09

**Authors:** Prince Zogli, Sophie Alvarez, Michael J. Naldrett, Nathan A. Palmer, Kyle G. Koch, Lise Pingault, Jeffrey D. Bradshaw, Paul Twigg, Tiffany M. Heng-Moss, Joe Louis, Gautam Sarath

**Affiliations:** 1 Department of Entomology, University of Nebraska-Lincoln, Lincoln, NE 68583-0816 USA; 2 Proteomics and Metabolomics Facility, Nebraska Center for Biotechnology, University of Nebraska-Lincoln, Lincoln, NE 68588 USA; 3 Wheat, Sorghum, and Forage Research Unit, USDA-ARS, Lincoln, NE 68583-0937 USA; 4 Biology Department, University of Nebraska-Kearney, Kearney, NE 68849 USA; 5 Department of Biochemistry, University of Nebraska-Lincoln, Lincoln, NE 68583-0816 USA

**Keywords:** Molecular biology, Proteomics, Biotic

## Abstract

Switchgrass (*Panicum virgatum* L.) is an important crop for biofuel production but it also serves as host for greenbugs (*Schizaphis graminum* Rondani; GB). Although transcriptomic studies have been done to infer the molecular mechanisms of plant defense against GB, little is known about the effect of GB infestation on the switchgrass protein expression and phosphorylation regulation. The global response of the switchgrass cultivar Summer proteome and phosphoproteome was monitored by label-free proteomics shotgun in GB-infested and uninfested control plants at 10 days post infestation. Peptides matching a total of 3,594 proteins were identified and 429 were differentially expressed proteins in GB-infested plants relative to uninfested control plants. Among these, 291 and 138 were up and downregulated by GB infestation, respectively. Phosphoproteome analysis identified 310 differentially phosphorylated proteins (DP) from 350 phosphopeptides with a total of 399 phosphorylated sites. These phosphopeptides had more serine phosphorylated residues (79%), compared to threonine phosphorylated sites (21%). Overall, KEGG pathway analysis revealed that GB feeding led to the enriched accumulation of proteins important for biosynthesis of plant defense secondary metabolites and repressed the accumulation of proteins involved in photosynthesis. Interestingly, defense modulators such as terpene synthase, papain-like cysteine protease, serine carboxypeptidase, and lipoxygenase2 were upregulated at the proteome level, corroborating previously published transcriptomic data.

## Introduction

Cereal aphids can successfully colonize and damage switchgrass (*Panicum virgatum*) plants^1^. Among the aphids tested, greenbugs (*Schizaphis graminum*, GB) caused significant plant damage likely through a combination of aphid-salivary proteins that are injected into plants during feeding and a strong host response elicited by herbivory^2,3^.

Plant resistance to herbivory has been broadly classified as antixenosis (deters insect settling), antibiosis (curtails insect fecundity), and tolerance^4,5^. Tolerant plants have compensatory mechanisms that allow for continued plant development with minimal yield losses, yet do not affect the fitness of the aphid^6^. Tolerance provides a reasonable means for improving plants in the face of continued pest pressure and are unlikely to select for the development of more virulent or resistant insect biotypes^4^.

An evaluation of switchgrass plants for categories of resistance indicated that plants of upland cultivar Summer were moderately susceptible to GB herbivory, but also demonstrated responses consistent with tolerance^7^; however, GB could not colonize or inflict significant damage on the lowland cultivar Kanlow^8,9^. Subsequently, a time course study of the changes in select metabolites and the transcriptomes of Summer switchgrass plants infested with GB was performed^3^. In this study, it was demonstrated that plant defensive metabolites, such as pipecolic acid, chlorogenic acid, and reactive oxygen species (ROS) were induced in response to GB herbivory. There were significant alterations to the transcriptomes of the infested plants with a peak in transcriptional changes occurring 10 days post aphid infestation (10 DPI). Furthermore, consistent with data reported in the literature^9,10^, there was a significant downregulation of genes associated with nutrient assimilation, photosynthetic pigment biosynthesis, and other growth-related pathways, and a concomitant upregulation of genes involved in plant defense and catabolic processes.

ROS and ROS mitigation are critical processes determining host responses to aphid herbivory^10,11^. Initial ROS signals arise from the respiratory burst oxidases (RBOHs) present on the plasma membrane^12^. Subsequent signaling cascades involves both apoplastic and symplastic propagation. Excess ROS produced by the host are mitigated by several classes of enzymes, such as peroxidases (PRX), catalases (CAT), superoxide dismutases (SOD), and glutathione-S-transferases (GST) among others. A failure to mitigate excess ROS has been linked to susceptibility to aphid herbivory in several plant species^13,14^. Upregulation of peroxidase genes and protein activity have been frequently linked to aphid herbivory as well as to tolerant or resistant plants^15^.

Several proteomic studies examining insect-plant interactions^16–21^ have been reported in the literature. Earlier studies had used a combination of 2-dimensional gel electrophoresis (2DGE) followed by mass spectrometry (MS), and some of the more recent reports have used other approaches such as tandem mass tag (TMT) of proteins followed by MS/MS^22,23^. The general trend shown in the literature suggests that besides upregulation of stress responsive proteins, insect feeding leads to changes in plant metabolism (carbohydrate, amino acid, and energy metabolism) and photosynthesis. As a consequence, genes involved in secondary metabolite biosynthesis and photosynthesis are up and downregulated, respectively^16,17^. Furthermore, increased oxidative stress response is accompanied by upregulation of proteins involved in detoxification^21^. Other studies reported high accumulation of enzymes involved in jasmonic acid (JA) and ethylene biosynthetic pathways, as well as serine proteases/protease inhibitors, in response to root herbivory^24^.

In this study, shotgun label-free proteomics^25^ has been used to document changes to the switchgrass proteome as a result of GB infestation. Additionally, changes in protein phosphorylation present in GB-infested and uninfested control plants was also determined. These proteomic data were compared against transcriptome changes recently published for this system^3^.

## Results

### Identification of differentially expressed proteins (DEPs)

To investigate the mechanisms of switchgrass responses against GB, the proteomic profiles of aphid-infested and uninfested control plants was performed on 10 DPI (Fig. S1). This time was chosen based on earlier data showing that peak transcriptomic responses occurred 10 DPI, with subsequent dampening in the response at 15 DPI^3^. Our goal was to capture as much differential details about the proteomes obtained from GB-infested and uninfested control plants and assess the extent to which GB infestation impacted the switchgrass proteome.

Peptide data generated in this study was used to search the switchgrass genome version 5.1 proteome database (https://phytozome-next.jgi.doe.gov/) and identified 3,594 proteins (with at least two unique peptides with high confidence, at 1% false discovery rate, Table S1). Overall, 429 of these proteins were identified as differentially expressed proteins (DEPs) with a log_2_ fold change Infected/Control (log_2_FC (I10/C10)) that are significantly different based on an adjusted *p* value ≤ 0.05 cutoff criteria (Table S2). A total of 291 and 138 of the 429 DEPs were up and downregulated, respectively, in response to GB herbivory (Table 1). The numbers of differentially expressed genes (DEGs) and the corresponding up and downregulated genes obtained from a previous transcriptomic study^3^ are shown for comparison in Tables 1 and S2.

**Table 1:** Total number loci identified and analyzed for this study.

	Significant loci/proteins identified	Upregulated	Downregulated
DEPs	429	291	138
DEGs^a^	10,032	6,174	3,858

*DEPs* differentially expressed proteins, *DEGs* differentially expressed genes.

^a^Previously published work^3^.

### GB infestation leads to upregulation of proteins involved in oxidative and secondary metabolic pathways, but suppresses proteins involved in photosynthesis and other related pathways

Enrichment analyses with GOBU^26^ revealed that upregulated proteins were significantly enriched with several functions related to chitinase activity and biosynthesis of secondary metabolites (Table 2). Proteins associated with secondary plant metabolism included several peroxidases, β-glucosidase family 13 proteins, cytochrome P450 proteins (Pavir.5KG587200, a homolog of AT2G40890 involved in lignin and flavonoid biosynthesis), a S-adenosyl-l-methionine-dependent cinnamyl-CoA-*O*-methyltransferase (CCoAOMT1, Pavir.6KG340400), and phenylalanine ammonia lyase 1 proteins (Table S2). Among other DEPs were two NAD(P)-binding Rossmann proteins, Pavir.7KG263500 and Pavir.7KG263200, whose homologs have been implicated in biosynthesis of defense-related terpenoids^27^. Similarly, three upregulated DEPs, Pavir.4KG114700, Pavir.9NG755900 and Pavir.6KG207900 were annotated as basic chitinases and homologs of Arabidopsis basic chitinase/PR3, which has been implicated as playing a role in the defense response of Arabidopsis^28^. Metabolic pathway enrichment also correlated well with biological process enrichment analysis and revealed an abundance of GO terms such as single-organism metabolism, oxidation–reduction process, response to stress, response to oxidative stress, and chitin catabolic/metabolic process (Table 2). Notably, protein domain analysis revealed a significant enrichment of NAD(P)-binding domain proteins and glutathione S-transferases (GST). The data included 14 upregulated NAD(P)-binding Rossman-fold proteins and 11 GSTs (Table S2), suggesting redox regulation as a critical component of switchgrass response to GB infestation. Arabidopsis homologs of three of the upregulated NAD(P) binding proteins, Pavir.9NG062049 (AT1G52340, ABA2), Pavir.7NG329400, (AT3G61220, SDR1) and Pavir.7KG263200 (AT2G24190, SDR2), are involved in resistance against microbial pathogens^29,30^.

**Table 2:** Enrichment analysis of significantly enriched PFAM domains, KEGG metabolic pathways, and molecular function GO terms among differentially expressed proteins (DEPs). Significant GO terms are reported here.

GO terms significantly enriched	# of GO terms present in reference genome	# of GO term hits among DEPs	*p* value	Adjusted *p* value
**Enrichment analysis of upregulated DEPs**				
Catalytic activity	14,617	146	3.63E−35	1.19E−32
Molecular function	27,777	190	1.35E−26	4.44E−24
Biological process	19,951	147	3.98E−21	2.22E−18
Metabolic process	15,661	125	5.41E−20	3.02E−17
Hydrolase activity	4,253	59	5.30E−19	1.74E−16
Oxidoreductase activity	3,021	46	2.33E−16	7.67E−14
Oxidation–reduction process	2,547	42	3.60E−16	2.01E−13
Hydrolase activity, hydrolyzing O-glycosyl compounds	730	22	5.84E−14	1.92E−11
Hydrolase activity, acting on glycosyl bonds	814	23	5.88E−14	1.93E−11
Cofactor binding	1,827	29	4.53E−11	1.49E−08
Carbohydrate metabolic process	1,098	20	5.45E−09	3.05E−06
Response to oxidative stress	336	11	5.43E−08	3.04E−05
Heme binding	961	17	1.18E−07	3.88E−05
Tetrapyrrole binding	964	17	1.23E−07	4.05E−05
Antioxidant activity	365	11	1.25E−07	4.11E−05
Response to stress	1,055	18	8.49E−08	4.75E−05
Binding	16,455	97	2.12E−07	6.97E−05
Chitinase activity	37	5	2.40E−07	7.90E−05
Metal ion binding	3,558	34	3.20E−07	0.000105
Cation binding	3,586	34	3.82E−07	0.000126
Glucosamine-containing compound catabolic process	37	5	2.40E−07	0.000134
Glucosamine-containing compound metabolic process	37	5	2.40E−07	0.000134
Amino sugar metabolic process	37	5	2.40E−07	0.000134
Aminoglycan catabolic process	37	5	2.40E−07	0.000134
Chitin catabolic process	37	5	2.40E−07	0.000134
Chitin metabolic process	37	5	2.40E−07	0.000134
Amino sugar catabolic process	37	5	2.40E−07	0.000134
Peroxidase activity	339	10	5.69E−07	0.000187
Oxidoreductase activity, acting on peroxide as acceptor	343	10	6.33E−07	0.000208
Drug catabolic process	41	5	4.07E−07	0.000228
Cell wall macromolecule catabolic process	41	5	4.07E−07	0.000228
Cell wall macromolecule metabolic process	46	5	7.34E−07	0.000410
Aminoglycan metabolic process	46	5	7.34E−07	0.000410
Oxidoreductase activity, acting on single donors with incorporation of molecular oxygen, incorporation of two atoms of oxygen	52	5	1.37E−06	0.000451
Dioxygenase activity	55	5	1.81E−06	0.000595
Carbohydrate derivative catabolic process	55	5	1.81E−06	0.001012
Response to stimulus	1,790	21	3.13E−06	0.001750
Oxidoreductase activity, acting on single donors with incorporation of molecular oxygen	70	5	6.04E−06	0.001987
Peptidase activity	1,134	15	2.12E−05	0.006975
Exopeptidase activity	152	6	2.14E−05	0.007041
Proteolysis	1,223	16	1.29E−05	0.007211
Magnesium ion binding	238	7	2.94E−05	0.009673
Lyase activity	431	9	3.28E−05	0.010791
Hydrolase activity, acting on acid phosphorus-nitrogen bonds	348	8	4.60E−05	0.015134
Serine-type peptidase activity	348	8	4.60E−05	0.015134
Serine hydrolase activity	348	8	4.60E−05	0.015134
Serine-type carboxypeptidase activity	111	5	5.66E−05	0.018621
Carboxypeptidase activity	113	5	6.16E−05	0.020266
Peptidase activity, acting on L-amino acid peptides	1,105	14	6.30E−05	0.020727
Terpene synthase activity	114	5	6.43E−05	0.021155
Cell wall organization or biogenesis	172	6	4.29E−05	0.023981
Serine-type exopeptidase activity	120	5	8.20E−05	0.026978
Carbon–oxygen lyase activity, acting on phosphates	121	5	8.53E−05	0.028064
Ion binding	8,249	51	0.00011	0.036848
**Enrichment analysis of downregulated DEPs**				
Photosynthesis, light harvesting	32	7	1.23E−13	6.88E−11
Catalytic activity	14,617	61	1.75E−12	5.76E−10
Photosynthesis, light reaction	56	7	8.22E−12	4.59E−09
Biological process	19,951	71	1.75E−11	9.78E−09
Photosynthesis	154	8	3.28E−10	1.83E−07
Generation of precursor metabolites and energy	233	9	3.47E−10	1.94E−07
Metabolic process	15,661	57	3.45E−09	1.93E−06
Carbohydrate metabolic process	1,098	12	5.08E−07	0.000284
Molecular function	27,777	75	1.57E−06	0.000517
Protein folding	193	6	1.18E−06	0.000660
Oxidoreductase activity	3,021	18	4.77E−06	0.001569
Hydrolase activity, hydrolyzing O-glycosyl compounds	730	8	4.23E−05	0.013917
Protein import	36	3	3.40E−05	0.019006
Oxidation–reduction process	2,547	15	3.56E−05	0.019900
Hydrolase activity, acting on glycosyl bonds	814	8	9.01E−05	0.029643

Consistent with pathway enrichment, upregulated proteins were enriched with molecular functions GO terms associated with catalysis, oxidoreductase activity, daphnetin 3-*O*-glucosyltransferase activity, and flavonol 3-O-glucosyltransferase activity (Table 2). Downregulated proteins in GB-infested plants were significantly enriched with biological process GO terms associated with photosynthetic and metabolic pathways (Table 2). All the downregulated proteins, such as Pavir.4KG305900, Pavir.5KG468900, Pavir.2NG555700 and Pavir.6KG271600, implicated in photosynthesis are involved with the Photosystem I light harvesting complex. Several other proteins linked to chloroplast function, such as albino or glassy yellow1, phytoene desaturase, thioredoxins, and uroporphyrinogen-III synthase, suggest a loss in plastid functions. Proteins linked to sucrose metabolism, such as sucrose phosphate synthase (SPSS) and a protein phosphatase that can dephosphorylate SPSS (BRI1 suppressor 1-like 2), were downregulated, indicating changes in sucrose biosynthesis (Table S2). Though downregulated proteins were also enriched with proteins having catalytic activity, as seen for the upregulated protein set, there was enrichment in chlorophyll binding and pigment binding, categories not enriched in GB-induced up-regulated proteins The group of proteins implicated in catalytic activity for the upregulated DEPs in response to GB herbivory were Pavir.6KG340400: CCoAMT1; Pavir.9NG661700: ALD1; Pavir.9KG072900: ornithine-delta-aminotransferase; and Pavir.3NG211100: terpenoid cyclase and downregulated proteins were Pavir.1NG556800: cytochrome P450; Pavir.5NG345600: peroxidase family protein; and Pavir.1KG250105: terpene synthase, suggesting that aphid attack in switchgrass remodels switchgrass metabolism.

### Identification of phosphorylated sites and their abundance changes

Protein phosphorylation is important for plant defense signaling^31,32^. To explore the roles of protein phosphorylation in switchgrass defense signaling, the phosphoproteome of switchgrass at 10 DPI was profiled using LC–MS/MS after phosphoenrichment of the same protein extracts used for proteomics analysis. A total of 2,044 phosphopeptides matching 996 proteins were identified (Table S3) with high confidence (< 1% peptide false discovery rate). Amongst these phosphopetides identified, 1,786 of them carried a single phosphorylation, 229 with two phosphorylation and 29 with three or more phosphorylation. The overlap in phosphosites between phosphopeptides is shown in Fig. S2. The number of unique phosphosites from the singly, doubly or triply and more phosphorylated peptides was 1,455, 398 and 74, respectively (Fig. S2). The total number of unique phosphosites identified and quantified here is 1,779. Amongst the 996 phosphoproteins, 310 were identified with a differentially phosphorylated (DP) site at 10 DPI with GB using following restrictions: (1) phosphopeptide detected in all four biological replicates, (2) adjusted *p* value < 0.05 and (3)|log_2_ FC (I10/C10)|> 1 (Table S4). Because of the allotetraploid nature of the switchgrass genome, if a phosphopeptide is associated with two or more potentially homeologous genes, all genes associated with significant phosphopeptides have been used for the analysis. This implies that a same phosphopeptide can be present more than once in the data set. These 310 DPs were identified with 350 phosphopeptides, which in total had 399 phosphosites (Table S5).

**Figure 1:**
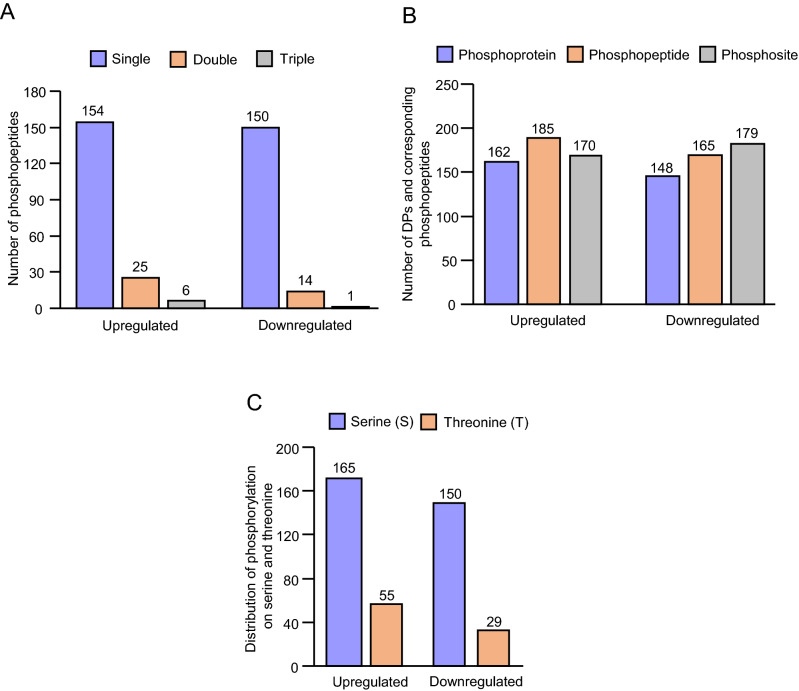
LC–MS/MS identification for phosphorylation sites of differentially phosphorylated proteins (DPs) from greenbug (GB)-infested switchgrass leaves. Leaf tissues were collected after 10 days post infestation of GB on switchgrass. (**A**) Distribution of single- and multi-phosphorylated peptides among DPs. (**B**) Number of phosphoproteins, phosphopeptides and phosphosites identified in the analysis. (**C**) The distribution of phosphorylated residues on serine (S) and threonine (T).

Figure 1A shows the distribution of the phosphopeptides up and downregulated identified with one, two or three phosphosites. About 87% of the phosphopeptides only have one phosphosite and 87% of the proteins with only one phosphopetide identified as DP. Among the 350 phosphopeptides, 185 had a significant increase in phosphorylation level, while 165 had a significant decrease in phosphorylation level in response to GB herbivory (Fig. 1B). The phosphorylated sites associated with the DPs are represented by 315 phosphoserines (79%) and 84 phosphothreonines (21%) (Fig. 1C). Interestingly, we identified a subset of 25 unique phosphopeptides associated with 12 proteins that displayed opposite phosphorylation abundance in response to GB herbivory (Table S6), which shows the importance of studying phosphorylation changes at each single phosphosite/phosphopeptide and not at the protein level. For example, among the three unique peptides associated Pavir.1KG036500, two had a significant increase in phosphorylation level while the other had a significant decrease in phosphorylation level (Table S6). Also, a cellulose synthase (Pavir.2NG127200) was identified with two peptides phosphorylated at positions S9 and S13 that were dephosphorylated and phosphorylated respectively, in response to GB herbivory. Other proteins with similar pattern of differentially phosphorylated or dephosphorylated residues included two ubiquitin-specific protease C19-related proteins (Pavir.6KG188500 and Pavir.6NG196600), two IQ-domain 14 proteins (Pavir.5KG696000 and Pavir.5NG012315), a RPM1 interacting protein4 (RIN4, Pavir.7KG167100), a hydroxyproline-rich glycoprotein (Pavir.2NG424700) and a calcium-binding EF hand protein (Pavir.4KG384303) (Table S6). These proteins may have very specific patterns of phosphorylation acting as switches in the regulation of defense mechanisms in switchgrass.

**Figure 2:**
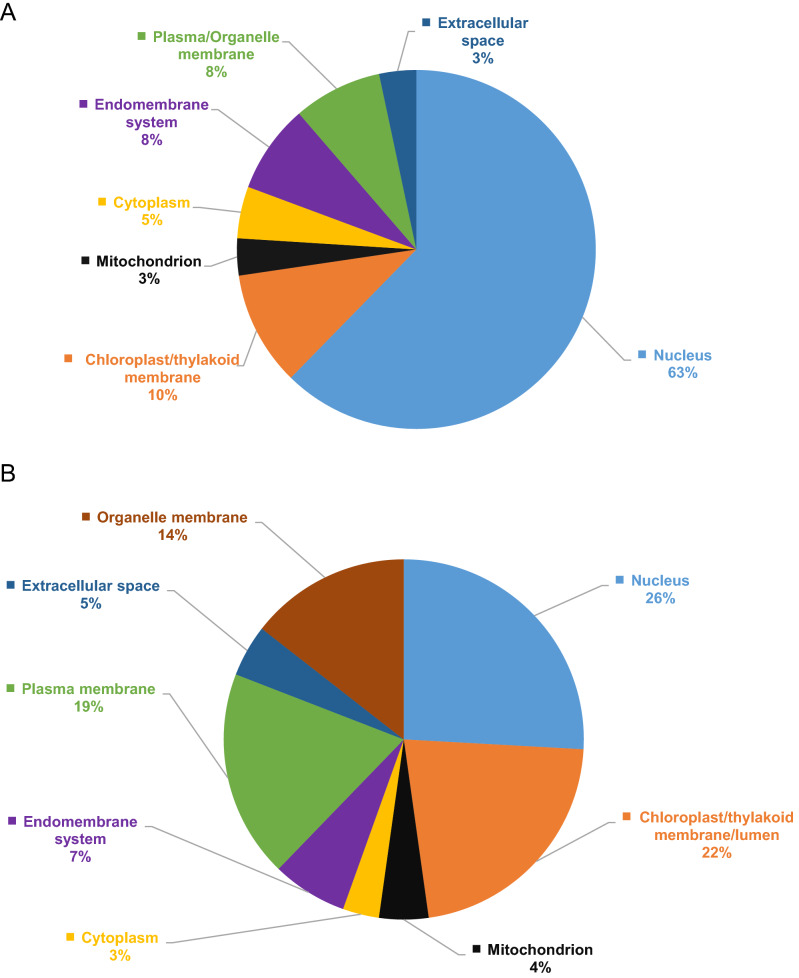
Subcellular localization of (**A**) DPs and (**B**) DEPs identified in switchgrass-greenbug interactions. BUSCA tool (https://busca.biocomp.unibo.it/) was used to analyze the subcellular localization of DPs and DEPs.

Changes to the subcellular partitioning of proteins is an essential part of plant defense responses, as different proteins need to be shuttled to specific cellular compartments to enact their functions, for example, cell wall fortification, redirection of primary metabolism, induction of gene expression, and protection of organelles from toxic byproducts to name just a few. In non-model plants such as switchgrass, these analyses also provide a means to comparing similar datasets with other more well characterized systems. BUSCA-based analysis (https://busca.biocomp.unibo.it/) showed that 63%, 10%, 8%, 8%, and 5% of the 310 DPs were located in the nucleus, chloroplast, endomembrane system, plasma/organelle membrane, and cytoplasm, respectively (Fig. 2A). The remaining 6% were shared equally between mitochondria and the extracellular space (Fig. 2A). Similarly, 26%, 22%, 19%, 14%, 7%, 5%, 4%, and 3%, of the 429 DEPs were located in the nucleus, chloroplast, plasma membrane, organelle membrane, endomembrane system, extracellular space, mitochondria, and cytoplasm, respectively (Fig. 2B).

### The multi-omics analysis shows correlations between gene regulation, protein abundance and phosphorylation changes in switchgrass upon GB infestation

A previous study at the transcriptomic level showed that GB infestation activates cellular oxidative responsive pathways and suppresses photosynthesis and other related pathways^3^. As we observed a similar trend at the proteomic level in the current study, the proteomic data was compared to the 10,032 differentially expressed genes (DEGs) reported previously^3^. These included genes that were both up and downregulated as a consequence of GB herbivory^3^. Out of 6,018 upregulated DEGs, 114 were also detected as upregulated DEPs, while 31 out of the 3,858 downregulated DEGs were reported as downregulated DEPs (Tables 3 and S7, Fig. S3A).

**Table 3:** Number of differentially expressed genes (DEGs), differentially expressed proteins (DEPs), and differentially phosphorylated proteins (DPs) identified in a comparative study.

	DEGs-up					
DEGs-up	6,018	DEGs-down				
DEGs-down		3,777	DEPs-up			
DEPs-up	114	19	154	DEPs-down		
DEPs-down	9	31		97	DPs-up	
DPs-up	25	13	4	NA	120	DPs-down
DPs-down	8	18	NA	1		120

Consistently, the group of genes upregulated at both transcript and proteome levels were enriched with proteins involved in secondary metabolite biosynthesis and stress/defense responses. These categories included Pavir.9NG661700 (ALD1), which is required for the biosynthesis of pipecolic acid, a key defensive compound that is significantly enhanced in response to GB herbivory, Pavir.6NG264600 (CCoAMT1) which participates in the biosynthesis of phenylpropanoid intermediates, Pavir.6KG367200 (LOX2) involved in oxylipin biosynthesis, Pavir.6NG135600 a mono/ sesquiterpene synthase that responds to herbivory^33^, Pavir.5KG357900 a glutathione S-transferase that is homologous to AT1G10360 (ERD9) that has been implicated in drought and stress tolerance in Arabidopsis^34^, and Pavir.2NG156200 (cytochrome P450 family protein, CYP99A3 family) potentially involved in the biosynthesis of phytoalexins and is also part of grass-specific family of proteins. Other defense genes like basic chitinases, PR3 Pavir.4KG114700 (AT3G12500) and PR4, Pavir.8KG305700 (AT3G04720) were also upregulated at both transcript and proteome level. Similarly, the group of genes downregulated at both transcriptome and proteome levels in response to GB were enriched with proteins such as Pavir.4KG305900, Pavir.6KG271600 and Pavir.2NG555700, which are chlorophyll a/b binding proteins and are involved in photosynthesis.

Overall, 429 DEPs in response to GB herbivory were identified, of which four upregulated DEPs were significantly increased in phosphorylation, while one downregulated DEP was dephosphorylated (Fig. S3B). In comparison, 64 DEGs were identified as differentially phosphorylated proteins (DPs) in response to GB infestation (Table 3). Among them, 25-upregulated and 18-downregulated DEGs also showed an increase and decrease in phosphorylation levels, respectively, upon GB infestation. The remaining 21 DEGs show an opposite response as follows: eight were upregulated DEGs but with a decrease in phosphorylation, and 13 were downregulated DEGs but significantly phosphorylated in response to GB herbivory (Table 4).

**Table 4:** List of previously reported DEGs significantly phosphorylated in this study.

Gene ID	Best hit Arabidopsis	Function	Phospho-peptide sequence and phosphorylated residues (in bold)	Category
Pavir.1KG096400	NA	NA	KVP**S**RPP**S**AHGHGHAPAPAPK	DEGs-up DPs-down
Pavir.1KG181862	AT1G53310	Phosphoenolpyruvate carboxylase 1	IRDPAFQV**S**PQPALSK	DEGs-up DPs-up
Pavir.1KG219100	AT2G36460	Aldolase superfamily protein	^1^GILAADE**S**TGTIGKR	DEGs-up DPs-up
Pavir.1KG438510	AT1G20440	Cold-regulated 47	EKLPGGHKKPEDAAAPAVHAPAPAPHAEDVG*S*PDGK	DEGs-up DPs-up
Pavir.1KG456400	AT4G13940	S-adenosyl-l-homocysteine hydrolase	^1^L**T**KSQADYISVPIEGPYKPAHYR	DEGs-up DPs-up
Pavir.1NG430900	AT4G13940	S-adenosyl-l-homocysteine hydrolase	^1^L**T**KSQADYISVPIEGPYKPAHYR	DEGs-up DPs-up
Pavir.2KG411435	AT2G33620	AT hook motif DNA-binding family protein	VAPAAPS**S**PPSR	DEGs-up DPs-up
Pavir.2KG484600	AT4G05150	Octicosapeptide/Phox/Bem1p family protein	SDAAE**T**PRQHGDEDEASVPAR	DEGs-up DPs-down
Pavir.2KG572300	AT1G53050	Protein kinase superfamily protein	IADFGLASFFDPNHKQPM**T**SR	DEGs-up DPs-up
Pavir.2NG515300	AT1G66950	Pleiotropic drug resistance 11	^1^WAAIEKLP**T**YDR	DEGs-up DPs-up
Pavir.2NG515300	AT1G66950	Pleiotropic drug resistance 11	SWLSAA**S**ISR	DEGs-up DPs-up
Pavir.3KG124322	AT4G09160	Sec14 cytosolic factor family protein/phosphoglyceride transfer family protein	AAEAD**S**EEEKKAEEALEAAAGDEAAVIDGAGSFK	DEGs-up DPs-up
Pavir.3KG261700	AT4G15530	Pyruvate orthophosphate dikinase	^1^**S**DFEGIFR	DEG-down DPs-down
Pavir.3KG310400	AT1G75220	Major facilitator superfamily protein	AGGAGYE**S**GSDHDGALQKPLLPNSGSWYR	DEG-down DPs-down
Pavir.3KG402302	AT1G15140	FAD/NAD(P)-binding oxidoreductase	VVQLTQQFQQ**S**FLEQNLGEK	DEG-down DPs-up
Pavir.3NG065800	AT1G75240	Homeobox protein 33	VHLVGDPEHLGQLGGGMPLPEPGGPGR**S**P**S**PSR**S**PPPQQLR	DEG-down DPs-up
Pavir.3NG066600	AT1G33800	Protein of unknown function (DUF579)	SSS**S**PMHAR	DEG-down DPs-down
Pavir.3NG066600	AT1G33800	Protein of unknown function (DUF579)	KAIHLA**S**LR	DEG-down DPs-down
Pavir.3NG076904	AT4G15530	Pyruvate orthophosphate dikinase	^1^**S**DFEGIFR	DEG-down DPs-down
Pavir.3NG183492	AT1G68830	STT7 homolog STN7	IVTTIKE**S**MDELTSQRK	DEG-down DPs-down
Pavir.3NG197700	AT1G15140	FAD/NAD(P)-binding oxidoreductase	AVQTQGAGVQ**T**QQGGAAR	DEG-down DPs-up
Pavir.3NG205200	AT3G48740	Nodulin MtN3 family protein	LPTTAAADEHVLVNIAKL**S**PALPEK	DEG-down DPs-down
Pavir.3NG212697	AT3G16630	P-loop containing nucleoside triphosphate hydrolases superfamily protein	AQNSANTQEEEKVTKV**S**PPR	DEGs-up DPs-down
Pavir.3NG233000	AT2G38280	AMP deaminase, putative / myoadenylate deaminase, putative	VAVIRPN**S**PK**S**PAASASAFE**S**VDGSDEDDATQHGGK	DEG-down DPs-up
Pavir.4KG382102	AT2G16860	GCIP-interacting family protein	RVVPAAADD**S**DEEAGAER	DEGs-up DPs-down
Pavir.4NG215687	AT2G42600	Phosphoenolpyruvate carboxylase 2	YGM**S**YIHETIWK	DEG-down DPs-down
Pavir.4NG215687	AT2G42600	Phosphoenolpyruvate carboxylase 2	DAGRL**S**AAWQLYR	DEG-down DPs-down
Pavir.4NG215687	AT2G42600	Phosphoenolpyruvate carboxylase 2	QLAPGKV**S**EDDKLVEYDVLLMER	DEG-down DPs-down
Pavir.5KG412607	AT1G15520	Pleiotropic drug resistance 12	^1^WAAIEKLP**T**YDR	DEGs-up DPs-up
Pavir.5KG453100	AT5G14740	Carbonic anhydrase 2	^1^LK**S**GFEQFK	DEG-down DPs-down
Pavir.5KG453100	AT5G14740	Carbonic anhydrase 2	^1^SGFEQFK**T**QVYDKKPELFEPLK	DEG-down DPs-down
Pavir.5KG466400	AT5G56580	MKK6	^1^FLTASG**T**FK	DEGs-up DPs-up
Pavir.5NG012556	AT2G36460	Aldolase superfamily protein	^1^GILAADE**S**TGTIGKR	DEGs-up DPs-up
Pavir.5NG016600	AT5G17380	Thiamine pyrophosphate dependent pyruvate decarboxylase family protein	ADSAASNP**S**PPNQKPLDEAIGK	DEGs-up DPs-up
Pavir.5NG178500	ATCG00710	Photosystem II reaction center protein H	A**T**Q**T**VEDSSRPKPK	DEG-down DPs-up
Pavir.5NG263800	AT5G14740	Carbonic anhydrase 2	^1^LK**S**GFEQFK	DEG-down DPs-down
Pavir.5NG263800	AT5G14740	Carbonic anhydrase 2	^1^SGFEQFK**T**QVYDKKPELFEPLK	DEG-down DPs-down
Pavir.5NG472800	AT5G56580	MKK6	^1^FLTASG**T**FK	DEGs-up DPs-up
Pavir.5NG577900	AT5G60010	Ferric reductase-like transmembrane component family protein	AQ**S**PGAGAGAGAGAGAGR	DEG-down DPs-up
Pavir.6KG023500	AT3G04120	Glyceraldehyde-3-phosphate dehydrogenase c subunit 1	HSDITLKD**S**KTLLFGEKPVTVFGIR	DEGs-up DPs-down
Pavir.6KG076000	AT1G34210	Somatic embryogenesis receptor-like kinase 2	LMDYKD**T**HV**T**TAVR	DEG-down DPs-up
Pavir.6KG276700	AT5G15210	Homeobox protein 30	VVHPGPSVASGAD**S**PL**S**A	DEG-down DPs-down
Pavir.6KG290606	AT1G56220	Dormancy/auxin associated family protein	KYA**S**FSPSSSSSLAPAAAPAVTR	DEG-down DPs-up
Pavir.6KG394700	AT4G38900	Basic-leucine zipper (bzip) transcription factor family protein	LNFAAGDE**S**PKLPLP**S**PGGGLTR	DEGs-up DPs-up
Pavir.6NG088100	NA	NA	NLGYTYDAE**S**EKELPWVASK	DEG-down DPs-down
Pavir.6NG092300	AT1G79040	Photosystem II subunit R	IK**T**DKPFGIGGGLTVDHDASGRK	DEG-down DPs-down
Pavir.6NG176100	AT5G15490	UDP-glucose 6-dehydrogenase family protein	DLAMNKFDWDHPMHLQPT**S**PSAVK	DEG-down DPs-down
Pavir.6NG271832	AT5G56000	Heat shock protein 81.4	TTEKEI**S**DDEDEEDKK	DEGs-up DPs-up
Pavir.7KG080900	AT5G58140	Phototropin 2	EIVEEPASSSPGAAAAGGGSYRQP**S**FQR	DEG-down DPs-down
Pavir.7KG080900	AT5G58140	Phototropin 2	SG**S**GGGKEIVEEPASSSPGAAAAGGGSYR	DEG-down DPs-down
Pavir.7KG182509	ATCG00120	ATP synthase subunit alpha	EAIQEQLERF**S**LQEQT	DEG-down DPs-down
Pavir.7KG182518	ATCG00120	ATP synthase subunit alpha	VINALAKPIDGRGEIVA**S**ESR	DEG-down DPs-down
Pavir.7KG182671	ATCG00020	Photosystem II reaction center protein A	**T**AILER	DEG-down DPs-up
Pavir.7KG306200	AT4G35310	Calmodulin-domain protein kinase 5	HAA**S**QRQDSEYSAADD**S**PKKPSTR	DEGs-up DPs-up
Pavir.7NG308700	AT4G35310	Calmodulin-domain protein kinase 5	AA**S**QRQDSEYSAAAADD**S**PKKPASR	DEGs-up DPs-up
Pavir.7NG390200	AT2G18960	H( +)-ATPase 1	GLDIDTIQQNY**T**V	DEGs-up DPs-up
Pavir.7NG401100	AT1G56080	NA	VTPGSTPMISSTGG**S**PR	DEGs-up DPs-up
Pavir.8NG016300	AT1G33110	Mate efflux family protein	**S**FISKDDDEQQVEEESSSLGR	DEGs-up DPs-up
Pavir.8NG106056	AT3G45780	Phototropin 1	RK**S**QEADCVFSTQVPGK	DEG-down DPs-up
Pavir.8NG194400	AT4G10340	Light harvesting complex of photosystem II 5	KPAQKPKPAAV**S**SSSPDISDELAK	DEG-down DPs-down
Pavir.9KG014300	AT3G25070	RPM1 interacting protein 4	SATQNDNKGDPETPSKDPP**S**AK	DEGs-up DPs-up
Pavir.9KG084000	AT5G04890	Hsp20-like chaperones superfamily protein	RP**S**LPRKP**S**AVEPPAPELPAR	DEGs-up DPs-up
Pavir.9KG385400	AT1G13740	ABI5 binding protein 2	TLGSLT**T**R	DEG-down DPs-up
Pavir.9KG394400	AT1G60420	DC1 domain-containing protein	FKV**S**GIPHLVILDAK	DEGs-up DPs-down
Pavir.9KG412083	AT1G59710	Protein of unknown function (DUF569)	LESSDSFSAPLHK	DEGs-up DPs-up
Pavir.9KG451600	AT2G41740	Villin 2	AAAVAALSNVLTAEG**S**HSPHHGRG**S**PTADAAK	DEG-down DPs-up
Pavir.9KG530300	AT2G02000	Glutamate decarboxylase 3	AV**S**ESDMSVHSTFASR	DEGs-up DPs-up
Pavir.9NG048300	AT3G29360	UDP-glucose 6-dehydrogenase family protein	DLAMNKFDWDHPMHLQPT**S**PTAVK	DEGs-up DPs-down
Pavir.9NG319500	AT2G03440	Nodulin-related protein 1	ELEPVPAAEEGK**S**EGFGLDDLVK	DEG-down DPs-down
Pavir.9NG472000	AT4G17330	G2484-1 protein	ASA**S**PEQQSVIASPQLK	DEG-down DPs-up
Pavir.9NG600700	AT2G39050	Hydroxyproline-rich glycoprotein family protein	ILPWGDEAYAGG**S**AANAPHGGHGHGEPTVR	DEGs-up DPs-down

NA indicate that the loci is not found in the reference dataset or category.

^1^Sequence represents items associated with more than one loci.

The group of genes upregulated at both transcript and proteome levels which also showed an increase in phosphorylation level in response to GB-feeding included proteins involved in plant-pathogen interactions, such as Pavir.9KG014300 (RIN4), Pavir.7KG306200 and Pavir.7NG308700 (CPK5), Pavir.5KG466400 and Pavir.5NG472800 (MKK6), and Pavir.6NG271832 (Hsp81.4). Conversely, downregulated DEGs that were decreased in phosphorylation level were enriched in proteins such as: Pavir.3NG183492 (STN7), Pavir.6NG092300 (PSBR), Pavir.4NG215687 (PPC2), Pavir.3NG076904 (PPDK), and Pavir.8NG194400 (LHCB5), which are involved in photosynthesis. A total of 13 transcription factors (TFs) were identified in this study only as DPs because these low abundance proteins, often regulated by phosphorylation, were enriched by the phosphoenrichment step in the phosphoproteomics study. Among the 13 TFs, two zinc-finger homeodomain proteins (Pavir.3NG065800 and Pavir.6KG276700) and a bZIP factor (Pavir.6KG394700), were transcriptionally up and downregulated respectively, in response to GB infestation. The remaining 10 TFs showed an increase in phosphorylation level only (Table S7), suggesting that these transcription factors are post-translationally regulated by phosphorylation in response to GB attack. These 10 TFs include a WRKY (Pavir.2NG560500), two calmodulin-binding transcription activators (Pavir.2KG546800, Pavir.9NG356600), one bHLH (MYC2; Pavir.9NG353828), and six zinc finger C3H TFs (Pavir.1NG017300, Pavir.2NG544400, Pavir.5NG172300, Pavir.7NG088447, Pavir.8KG143501 and Pavir.8NG213620).

## Discussion

Proteomics has emerged as a powerful tool to explore physiological changes at the cellular level^35,36^ and has been used to study plant defense responses to herbivory^19,22,23,37^. The present study is a comprehensive analysis of changes occurring to the switchgrass proteome and phosphoproteome in response to GB herbivory. Switchgrass cultivars have distinct responses to aphids^1^, and the cultivar Summer, though damaged by GB appeared to have a tolerant response. Plant damage, physiological and transcriptomic studies of Summer switchgrass responses to GB infestation^3^ largely corroborated these earlier phenotypic findings indicating that 10 DPI is a good sampling time to assess changes in the switchgrass proteome as a result of GB infestation. To our knowledge, this is the first study that utilizes proteomic and phosphoproteomic approaches to monitor switchgrass defense responses to GB herbivory. The two methods identified a total of 3,594 proteins and 2,044 phosphorylated peptides belonging to 996 proteins. GO enrichment and KEGG pathway analysis indicated that proteins involved in host plant processes such as secondary metabolite metabolism, redox regulation, and photosynthesis are significantly altered by GB infestation.

### DEPs involved in secondary metabolite biosynthesis

Secondary metabolites produced by plants can influence herbivore feeding on plant tissues^38^. Plants activate secondary metabolites as potential defense mechanisms against microbial and insect attacks. Several plant metabolites including alkaloids, terpenoids, isoflavonoids, oxoacids, carboxylic and benzoic acids negatively affects the performance of herbivores^39^. Oxoacid and carboxylic acid-dependent defense pathways against insects through glucosinolates and SA-dependent gene regulation were reported in the model plant Arabidopsis^40,41^. Evidence from maize indicates a role for the benzoxazinoid and related products of plant secondary metabolism in plant defense^42,43^. It is tempting to speculate that switchgrass would similarly synthesize secondary metabolites in response to insect stress. The present study uncovered differential regulation of several proteins involved in secondary metabolite biosynthesis. Proteins induced by GB infestation included enzymes essential for the biosynthesis of phenylpropanoid intermediates (CCoAMT1, Pavir.6NG264600)^44^, terpene biosynthesis, glutathione-related metabolism, and oxylipin biosynthesis. Elevated levels of terpene synthases are consistent with recent transcriptomic and biochemical data that indicated a significant upregulation of switchgrass terpene synthase encoding genes and increased enzymatic activities in response to feeding by aphids and caterpillars^3,33^. However, not all genes associated with specific aspects of secondary plant metabolism were induced by insect herbivory, suggesting role(s) in other aspects of plant metabolism.

Four lipoxygenases (LOXs), encoded by Pavir.6KG367200, Pavir.1KG101800 Pavir.6NG315500 and Pavir.3KG264209, were induced in response to GB. LOXs catalyze the oxidation of polyunsaturated fatty acids generating hydroperoxy fatty acids. The Arabidopsis homolog, *AtLOX2* (AT3G45140) encodes a 13(S)-lipoxygenase that controls the first dedicated step in the biosynthesis of JA^45^. JA is an important hormone induced in response to insect herbivory^46^. Therefore, the significant induction of LOX2 homologs suggest that switchgrass relies on LOX2-mediated JA signaling to activate their basal defense. In Arabidopsis, *At*LOX2 is constitutively phosphorylated at Ser_600_ and targeted by insect salivary effectors for dephosphorylation to lower JA accumulation^47^, thereby promoting herbivory. The *At*LOX2 homolog in switchgrass (Pavir.7KG108800) was significantly upregulated and phosphorylated in response to GB herbivory, which suggest a similar *At*LOX2-dependent JA signaling mechanism may exist in switchgrass to deter herbivory. However, a LOX family protein (Pavir.9NG150900), likely a LOX5 homolog, was downregulated in switchgrass after GB infestation. Root enhanced expression of Arabidopsis LOX5 positively impacts green peach aphid (*Myzus persicae*) feeding^48^. It is intriguing to speculate that downregulation of switchgrass LOX5 may have a negative influence on GB feeding. Taken together, the current proteomic, phosphoproteomic, and previous transcriptomic data^3^, indicate that JA biosynthesis is likely impacted via a complex regulatory network in switchgrass.

### DEPs involved in oxidative stress

In response to plant herbivores, reactive oxygen species (ROS) are generated in plant tissues^49^. Plant defense responses against aphids include calcium influxes^50^, accumulation of ROS^51^, phloem occlusion^52,53^, and callose deposition^54^. High and low accumulation or reduction of ROS make plants resistant or susceptible to aphids, respectively. For instance, the induction of ROS activity was very high in the resistant wheat (*Triticum aestivum*) infested with Russian wheat aphid (*Diuraphis noxia*), but low when infested with a more virulent *D. noxia*^55^. Also, GB feeding on resistant sorghum (*Sorghum bicolor*) genotype caused high expression of peroxidase leading to ROS production^56^. ROS is proposed to be produced by different enzymes, including NADPH oxidases^57^, peroxidases^58^, and oxalate oxidase^59^. However, though ROS are important signaling molecules in plants, high abundance can be toxic to the plant and ROS scavenging is critical for plant health. As such, high amounts of ROS can be removed by ROS-scavenging enzymes like catalases^60^, peroxidases^61^, and superoxide dismutases^62^, as well as antioxidants like ascorbate and glutathione^63^. Apart from scavenging, peroxidases are essential players in auxin catabolism, programmed cell death, defenses against pathogens, and cell wall lignification^64^. Oxidative stress-related proteins were differentially regulated upon GB infestation, and included peroxidases, catalases, superoxide dismutates, dioxygenases, and other reductases and GSTs. Interestingly, switchgrass oxidative stress proteins were upregulated in response to drought^65^, suggesting switchgrass may use similar pathways to combat biotic and abiotic stress. Pavir.8NG068900, a homolog of Arabidopsis α-dioxygenases (α-DOX1, AT3G01420), is an enzyme that may contribute to the synthesis of oxylipin, a signaling molecule implicated in plant defense against tobacco hornworm and aphids^66, 67^. It is highly likely that Pavir.8NG068900, like other α-DOXs, may play a similar role in conferring switchgrass resistance to GB herbivory.

### DEPs and DPs involved in stress response

Plants have constitutive and inducible protective mechanisms to overcome various biotic and abiotic stresses^68^. Pathogenesis-related proteins (PR proteins) and defense-related proteins are specifically induced under stress conditions^68^. In this study, several known stress response proteins were induced in switchgrass in response to GB herbivory. Among them were papain family cysteine proteases (Pavir.1KG224700), PR1 (Pavir.5KG293200), PR4s (Pavir.8NG270602, Pavir.8KG305700), disease resistance-responsive protein (Pavir.3KG066327), MLP-like protein (Pavir.3NG236300), and HOPW1-1-interacting2 (Pavir.7KG429900 and Pavir.3NG149165) proteins. Papain-like cysteine protease (PLCPs) are increasingly being reported as key players in plant immune signaling pathways^69^. In fact, a papain-like cysteine protease, Maize insect resistance1-Cysteine Protease (Mir1-CP) has been shown to provide direct toxicity to corn leaf aphid (*Rhopalosiphum maidis*)^70^. Similarly, *At*HOPW1-1-interacting proteins act as receptors to recognize *Pseudomonas syringae* effector HOPW1-1, leading to disease resistance in Arabidopsis^71^. It is therefore possible that Pavir.7KG429900 functions as a similar receptor for aphid-secreted effectors to activate resistance. However, another member of HOPW1-1-interacting protein family (Pavir.1NG023600) was downregulated after GB infestation, suggesting that effectors present in GB saliva may have evolved strategies to repress the function of this protein.

When comparing phosphoproteomic data generated in this study to previously published RNA-seq data^3^, 64 DPs were similarly regulated at the transcript level. Among those, 25 were significantly upregulated at the transcription and phosphorylation levels and phosphorylated in response to GB feeding (Table 4; see DEGs-up DPs-up category). These groups of proteins were enriched in proteins involved in plant-pathogen interaction pathway, including: Pavir.6NG271832 (heat shock protein), Pavir.6KG394700 (bZIP transcription factor), Pavir.5NG472800 (MAP kinase kinase6), Pavir.7KG306200 and Pavir.7NG308700 (homeologous pair calmodulin-domain protein kinases), and Pavir.9KG014300 (RPM1 interacting protein 4, RIN4). Arabidopsis RIN4 and its orthologs are conserved in land plants and are targeted and phosphorylated by *P. syringae* secreted virulence proteins^72^. Intriguingly, RIN4 phosphorylation and its specific protein–protein interaction can activate or suppress plant immune responses^72^. For example, AvrRpm1 was recently reported to act as ADP-ribosyl transferase that promotes phosphorylation of AtRIN4, thereby inhibiting secretion of defense compounds^73^. Though *At*RIN4 is phosphorylated at Thr-21, 166, and S20, Pavir.9KG014300 (*Pv*RIN4) identified in this study is phosphorylated at S54, suggesting that RIN4 may be regulated differently in herbivory defense compared to defense against bacteria. Therefore, it will be interesting to know whether switchgrass RIN4′s phosphorylation is also a mechanism utilized by aphid effectors to enhance virulence. Two uncharacterized DPs, Pavir.7NG401100 (NAI2-Interacting Protein 3, NAIP3) and Pavir.9KG412083 (actin cross-linking protein), were also reported as DEGs. Recent reports implicate NAIPs in the biogenesis of ER Bodies^74^. Because ER bodies are also known for providing defense against herbivory^75,76^, further investigation of the role of Pavir.7NG401100 in switchgrass defense to aphids is intriguing.

## Conclusions

In the present study, the proteomic and phosphoproteomic responses of switchgrass cultivar Summer to GB attack at 10 DPI was conducted. Previous research suggested that plant metabolism is altered during insect attack. In this study, we observed a global repression of photosynthesis, but upregulation of pathways involved in secondary metabolite biosynthesis. Besides changes in secondary metabolite biosynthesis, herbivory leads to numerous changes in plant primary metabolism as well^77^. Repression of photosynthesis is among the early responses to aphid herbivory, and the proteomic data are consistent with transcriptomic data published previously for switchgrass-GB interactions^3^. This suggest suppression of photosynthesis is a global response to biotic stress attacks, potentially to reduce the amount of nutrients available to the herbivore, redirect the movement of sucrose to distal sources, and recalibrate the sugars to starch ratios.

In addition, we found some correlation between regulation of protein abundance and protein phosphorylation in response to GB, proteins such as Pavir.7KG134400 (SNF1-related protein kinase), Pavir.7KG108800 (LOX2), and Pavir.2KG476205 (Eukaryotic translation initiation factor 4G) were both upregulated in protein abundance and phosphorylation. Our comparative analysis revealed that switchgrass homologs of Arabidopsis defense regulators such as PR1, terpene synthase, papain cysteine protease, serine carboxypeptidase, and LOX2 were upregulated at both transcript and proteome levels. The patterns of protein localization of the DEPs and DPs were similar, with majority being nuclear localized, followed by plastid, membrane, and organelle membrane localization. Chloroplast localization of several DEPs and DPs is intriguing, since they produce many defense-related molecules, including JA and secondary messengers such as ROS. Similarly, the nucleus acts as the propagation hub of pathogen or herbivore-induced hormonal signaling pathways, leading to changes in gene expression and defense response. Also the secretory pathway participates in plant defense through delivery of defense proteins and defensive secondary metabolites to the extracellular space, and mediating localized callose deposition^78^. Therefore the extensive targeting of GB-feeding induced DPs and DEPs to the nucleus, chloroplast, and endomembrane system further reiterate the importance of these compartments to the switchgrass immune response.

Furthermore, phosphorylation of proteins like Pavir.9NG353828, a homolog to *At*MYC2, may act as a key TF modulator of JA responses during plant defense^79^. Recently, rice (*Oryza sativa*) MYC2 was also reported to be an essential factor for JA-dependent production of sakuranetin^80^, a defense-related phytoalexin that accumulated in blast-infected rice leaves^81^. In the future, studies on the function of specific proteins found in this study, such as Pavir.9NG353828 (*Pv*MYC2), will be helpful to explore the mechanisms of host resistance to GB and other aphid pests of switchgrass.

## Materials and methods

### Plant material and treatments

Plants of switchgrass cultivar Summer were grown from seed in Containers (Ray Leach SC10; Stuewe & Sons, Inc, Tangent, OR) to the L2 stage^82^, under 400-W high intensity lamps with a 16:8 (light:dark) photoperiod and 23 ± 4 °C temperature in a greenhouse^1^. Fifty plants were randomly selected for this experiment. Plants were arranged in a 2 × 4 factorial design, which had two treatments (aphid infested and uninfested plants). Ten days post infestation (DPI) time point for leaf sample collection was selected based on previous transcriptomic data showing maximal upregulation of defense pathways 10 DPI^3^. Each plant was infested with 10 GB (biotype I) at day 0, after which each plant was caged with tubular plastic cages with vents covered with organdy fabric to restrict the aphid movement on the infested plants. Control plants (aphid uninfested) were similarly caged. Both aphid infested and control plants were kept in the greenhouse conditions described above for 10 days before leaf samples were taken. Aphids were removed from the leaves before sample collection. Leaves were flash frozen under liquid N_2_ conditions, ground to a fine powder using a mortar and pestle and were stored at − 80 °C, until analyzed.

### Protein extraction and digestion

Total protein was extracted from four biological replicates of two treatments: C10 (controls) and I10 (infested). Protein extraction from the ground leaf tissues were done as described previously by Alvarez et al.^83^. The protein pellet was briefly air-dried, then redissolved in a solution containing 7 M urea, 2 M thiourea, 5 mM DTT, containing 1x PhosSTOP phosphatase inhibitor (Roche, Basel, Switzerland) and 1x cOmplete, EDTA-free Protease Inhibitor Cocktail (Roche, Mannheim, Germany). Protein amounts were determined as previously described^84^. 800 µg of total protein from each sample was reduced and alkylated as previously described in Alvarez et al.^85^. Samples were diluted tenfold and trypsin digestion carried out for 24 h at a ratio of 1:50 enzyme: substrate (E:S). A further aliquot of trypsin (1:50 E:S) was added and digestion carried out for a further 3 h. Digests were acidified with 20% TFA to pH 3, then desalted using 50 mg Sep-Pak C18 reverse-phase SPE columns (Waters Corp, Milford, MA). A portion was set aside for analysis of the unenriched sample.

### Phosphoenrichment

Approximately, 0.75 mg of digested, desalted, dry peptide was dissolved in 2 M lactic acid, 60% acetonitrile to 3 mg/mL and shaken violently with TiO_2_ beads (Titansphere, 5 µm, GL Sciences, Tokyo, Japan) in a ratio of 1:4 sample:beads (w/w) for 1 h at 24 °C as described previously^86^. The suspended beads were then placed into a 200 µL tip (Eppendorf, Hauppauge, NY) plugged with 2 layers of 3 M C8 Empore membrane (Thermo Fisher Scientific, USA). 100 µL of the same solution was spun through the beads at 3,000×*g* three times. The beads were then further washed with 3 × 100 µL of 80% acetonitrile at 3,000×*g*. Phosphopeptides were eluted into 1.5 mL Lo-Bind tubes (Eppendorf, Hauppauge, NY) by 2 × 100 µL additions of ammonium hydroxide (5% v/v) at 1,000×*g*, frozen and immediately lyophilized. A further elution with 2 × 100 µL pyrrolidine (5% v/v) at 1,000×*g* was performed, and this pooled eluate was frozen and immediately lyophilized. Both eluates were combined and analyzed by LC–MS/MS.

### LC–MS/MS analysis of the proteome and phosphoproteome

The eight proteomic samples and the eight samples enriched for phosphopeptides were analysed by LC–MS/MS on an RSLCnano system (Thermo Fisher Scientific, USA) coupled to a Q-Exactive HF mass spectrometer (Thermo Fisher Scientific, USA). The samples were first injected onto a cartridge trap column (PepMap 100, C18, 0.3 × 5 mm, Thermo Fisher Scientific, USA) for 3.3 min at a flow rate of 5 µL/min, 2% acetonitrile, 0.1% formic acid before switching in line with the main column. Separation was performed on a C18 nano column (ACQUITY UPLC M-class, Peptide CSH 130A, 1.7 µm 75 µm × 250 mm, Waters Corp, Milford, MA) at 260 nL/min with a linear gradient from 5–35% over 96 min. The LC mobile phases were as follow: A contained 0.1% (v/v) formic acid in water and B contained 0.1% (v/v) formic acid in 80% (v/v) acetonitrile. Mass spectra for the eluted peptides were acquired on a Q Exactive HF mass spectrometer in data-dependent mode using a mass range of *m/z* 375–1,500, resolution 120,000, AGC target 3 × 10^6^, maximum injection time 60 ms for the MS1 peptide measurements. Data-dependent MS2 spectra were acquired by HCD as a Top20 experiment with a normalized collision energy (NCE) set at 28%, AGC target set to 1 × 10^5^, 15,000 resolution, intensity threshold 1 × 10^4^ and a maximum injection time of 250 ms. Dynamic exclusion was set at 20 s to help capture phospho isomers and the isolation window set to 1.6 m*/z*.

### Data analysis

Data were analyzed in Proteome Discoverer 2.2 software (Thermo Fisher Scientific, USA). Three different database search tools were used, Mascot 2.6.2, MS Amanda 2.0 and SeQuest HT. The databases searched were the common contaminants database cRAP (116 entries, www.theGPM.org) and the Pvi5 (79,335 entries, www.phytozome.org). For the proteomics experiment, methionine oxidation, protein N-terminal and lysine acetylation, methylation, dimethylation and trimethylation of arginine and lysine were set as variable modifications, whilst Cys carbamidomethylation was specified as a fixed modification. For the phosphoproteomics experiment, methionine oxidation, protein N-terminal and lysine acetylation, and Ser/Thr and Tyr phosphorylation were set as variable modifications, whilst Cys carbamidomethylation was specified as a fixed modification. The search included a maximum of two trypsin missed cleavages with the precursor mass tolerance set to 10 ppm and the fragment mass tolerance to 0.02 Da, respectively. Peptide validation were done by Percolator with a 0.01 posterior error probability (PEP) threshold. The data were searched using a decoy database to set the false discovery rate to 1% (high confidence). The localization probabilities of the PTMs were obtained using ptmRS^87^. The peptides were quantified using the precursor abundance based on intensity. The peak abundance was normalized for differences in sample loading using total peptide amount where the peptide group abundances were summed for each sample and the maximum sum across all runs was determined. The normalization factor used was the factor of the sum of the sample and the maximum sum in all files. The protein ratios, expressed as log_2_ fold change (Infected/Control), or log_2_FC (I10/C10), were calculated using summed of the peptides abundance for each sample and replicate separately. The geometric median from the four replicates was used to calculate the protein ratios. To compensate for missing values in some of the replicates, the replicate-based resampling imputation mode was selected. The significance of differential expression was tested using an ANOVA test, which provides adjusted p-values using the Benjamini–Hochberg method for all the calculated ratios, based on the summed abundances from the four replicates. For the phosphoproteomic analysis, data was filtered further to remove phosphopeptides with phosphosites not confidently localized (score < 95% according to ptmRS). The quantitative analysis was done at the phosphopeptide level and not at the phosphoprotein level for a better representation of the phosphorylation abundance change for each protein. Phosphosites identified to more than one gene ID were counted several times when reporting the proteins differentially phosphorylated.

### Integration of proteomic, transcriptomic and phosphopeptides data

The integration between the transcriptomic, proteomic and phosphopeptide data is presented in the Table S7. The transcriptome data used in this study was previously published^3^. We retained only the information collected at 10 days for both aphid infested and uninfested controls. Genes with the FC >|2| and adjusted *p* value < 0.05 were identified as differentially expressed genes (DEGs). For the protein abundance and phosphoprotein, differentially expressed proteins (DEPs) and differentially phosphorylated-sites (DPs) were identified by adjusted *p* value < 0.05.

### Functional annotation

The GOBU package was used for enrichment calculations^26^. The full set of switchgrass gene annotation was used as the reference comparison set against down or upregulated DEPs. The *p* values were calculated using Fisher’s exact test and corrected for multiple testing with FDR method using the R module called ‘p-adjust’.

## Supplementary information

Supplementary information

Supplementary table S1

Supplementary table S2

Supplementary table S3

Supplementary table S4

Supplementary table S5

Supplementary table S6

Supplementary table S7

## Data Availability

Proteomic data have been deposited under https://proteomecentral.proteomexchange.org/cgi/GetDataset. The temporary numbers are 1–20200410-130072 for the proteomics study and 1–20200410-77144 for the phosphoproteomics study. The RNA-Seq datasets utilized in this study are available in the SRA repository, Accession number SRX1600826. Other datasets generated and/or analyzed during the current study are available from the corresponding authors on reasonable request.
