# Supplementary material for: Greenbug (Schizaphis graminum) herbivory significantly impacts protein and phosphorylation abundance in switchgrass (Panicum virgatum)
Source: Sci Rep. 2020 Sep 9;10:14842. doi: 10.1038/s41598-020-71828-8 (PMC7481182; doi:10.1038/s41598-020-71828-8)
Supplement: Supplementary file 1 — Supplementary information [file 41598_2020_71828_MOESM1_ESM.pdf]

Supplementary information

Greenbug (*Schizaphis graminum*) Herbivory Significantly Impacts Protein and Phosphorylation Abundance in Switchgrass (*Panicum virgatum*)

Prince Zogli[1,a], Sophie Alvarez[2,a], Michael J. Naldrett[2,a], Nathan A. Palmer[3,a], Kyle G. Koch[1], Lise Pingault[1], Jeffrey D. Bradshaw[1], Paul Twigg[4], Tiffany M. Heng-Moss[1], Joe Louis[1,5,*], Gautam Sarath[1,3,*]

[1]Department of Entomology, University of Nebraska-Lincoln, Lincoln, NE 68583
[2]Proteomics and Metabolomics Facility, Nebraska Center for Biotechnology, University of Nebraska-Lincoln, Lincoln, NE 68588
[3]Wheat, Sorghum, and Forage Research Unit, USDA-ARS, Lincoln, NE 68583
[4]Biology Department, University of Nebraska-Kearney, Kearney, NE 68849
[5]Department of Biochemistry, University of Nebraska-Lincoln, Lincoln, NE 68583

[a]Authors contributed equally to this study

*Corresponding authors:

Joe Louis
Department of Entomology &
Department of Biochemistry
212 Entomology Hall
University of Nebraska–Lincoln
Lincoln, NE 68583-0816
(402) 472-8098
joelouis@unl.edu

Gautam Sarath
Wheat, Sorghum, and Forage Research Unit
USDA-ARS
251 Filley Hall
University of Nebraska-Lincoln
Lincoln, NE 68583-0937
(402) 472-4204
Gautam.Sarath@ars.usda.gov

Zogli et al.
Figure S1

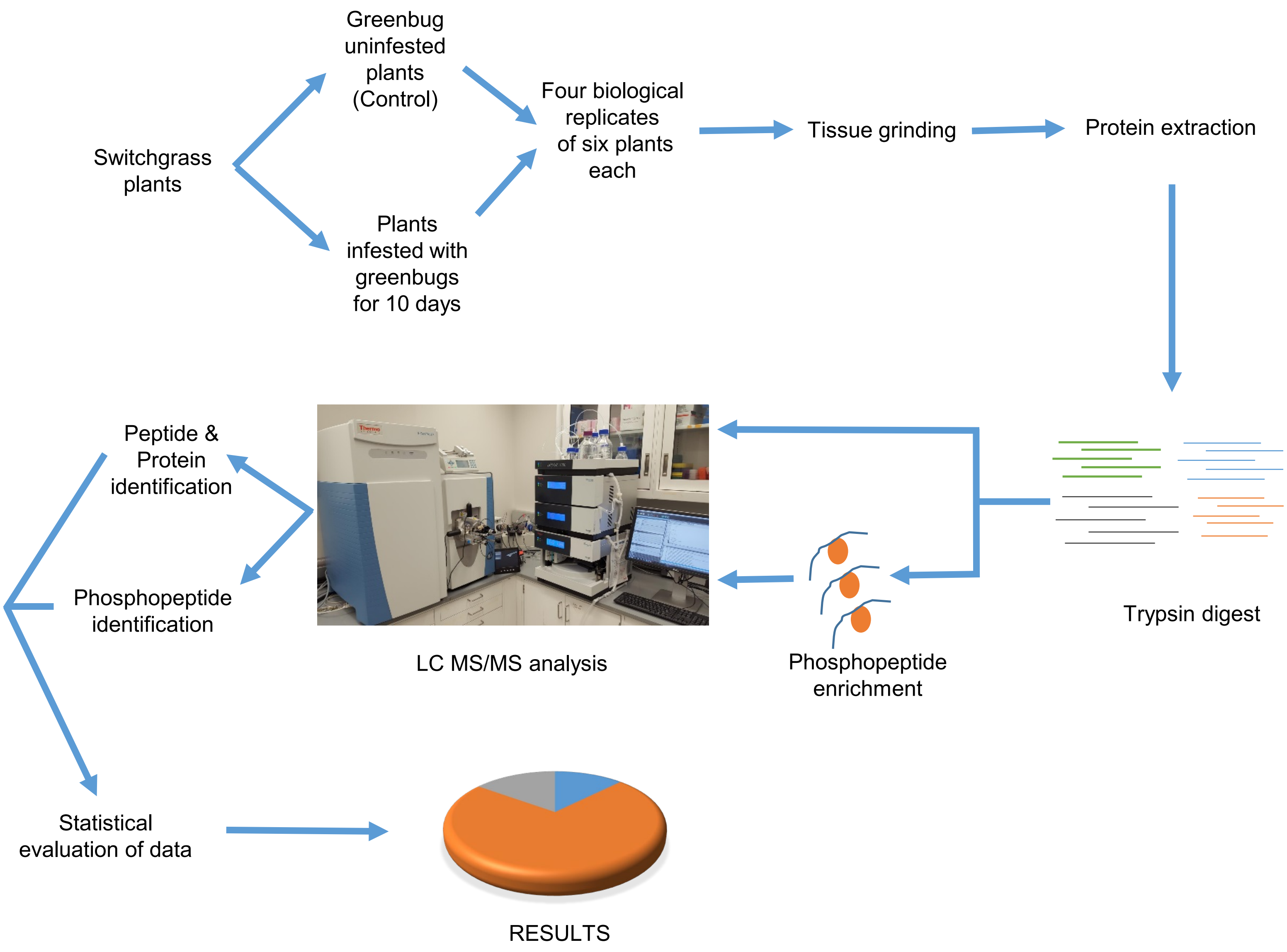

Figure S1: Overview of proteomics pipeline for analysis of differential expression of proteins and phospho-peptide identification in switchgrass 10 days post greenbug (GB) infestation. Ten GB were initially placed on infested plants after which plants were individually caged with tubular plastic cages with vents to confine GB on the infested plants. Uninfested plants served as control. Infested leaves were harvested from six switchgrass plants for protein extraction upon grinding to powder using the Geno/grinder. Protein extracts were subjected to trypsin digestion followed by phosphoenrichment before protein and phosphopeptide identification using LC-MS. Four biological replicates were used to quantify protein abundance and degree of phosphorylation upon GB infestation. LC MS/MS platform picture taken by Sophie Alvarez.

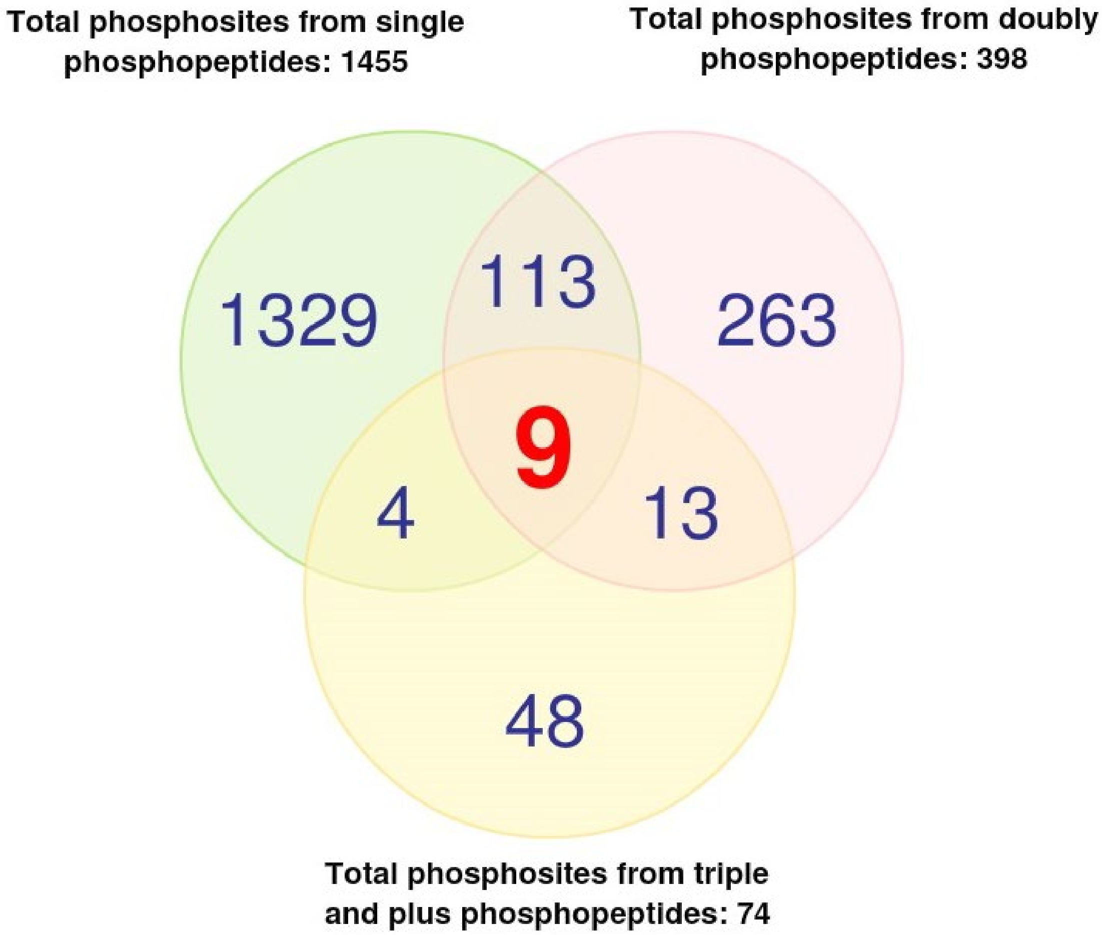

Figure S2: Representation of LC-MS/MS quantification of the phosphoproteome of switchgrass 10 days after GB infestation. Venn diagram shows the overlap in phosphosites among phosphopeptides identified with high confidence (<1% peptide false discovery rate) as found in Table S3.

Zogli et al.
Figure S3

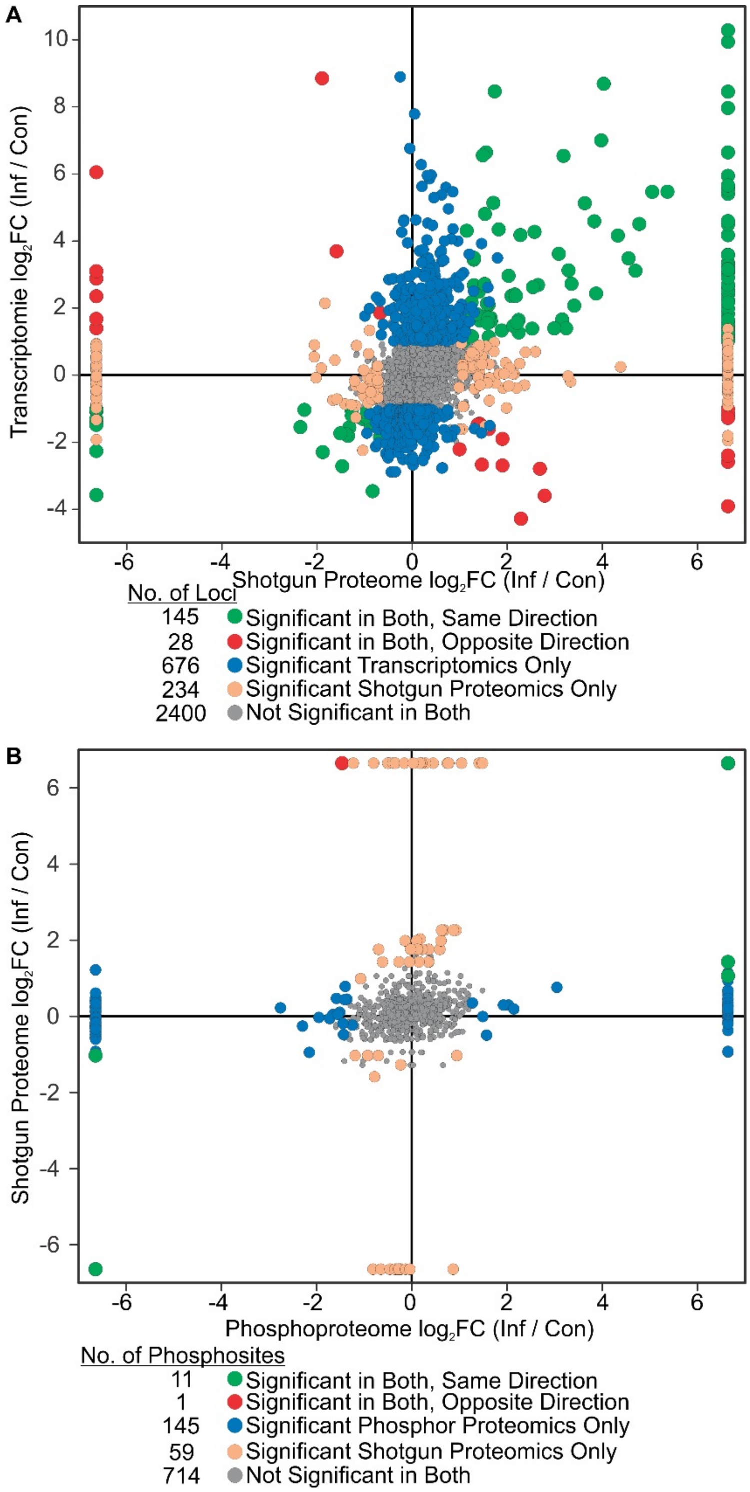

Figure S3: Graphical representation of relationship between DEGs, DEPs and DPs. (A) Comparative studies of shotgun proteomic data and published RNA-Seq experiment, plotting the $\log_2$ FC (Infested / Control) for the proteomics on the X-axis and the $\log_2$ FC (infested / Control) for the RNA-Seq on the Y-axis. Each point is a separate locus (3483 total), with colors described in the legend. (B) Comparison of the shotgun proteomic results to the phosphor-proteomics data. The X-axis is the $\log_2$ FC (Infested / Control) for the phosphoproteomics and the Y-axis is the $\log_2$ FC (Infested / Control) from the shotgun proteomics. In this plot, each point is a phosphosite (930 phosphosites on 349 total loci). In some cases, phosphosites were assigned to multiple loci. In these cases, the phosphosite results were paired with each individual locus.
